# Supplementary material for: Drainage explains soil liquefaction beyond the earthquake near-field
Source: Nat Commun. 2023 Sep 27;14:5791. doi: 10.1038/s41467-023-41405-4 (PMC10533503; doi:10.1038/s41467-023-41405-4)
Supplement: Supplementary file 1 — Supplementary Information [file 41467_2023_41405_MOESM1_ESM.pdf]

Supplementary Information
Drainage explains soil liquefaction beyond the earthquake near-field

Shahar Ben-Zeev[1,2,*], Liran Goren[3], Renaud Toussaint[2,4], and Einat Aharonov[1,5]

[1]Institute of Earth Sciences, The Hebrew University of Jerusalem, 91904, Israel
[2]Université de Strasbourg, CNRS, ENGEES, Institut Terre & Environnement de Strasbourg, UMR7063, F-67000 Strasbourg, France
[3]The Department of Earth and Environmental Sciences, Ben-Gurion University of the Negev, 84105, Israel
[4]PoreLab, the Njord Centre, Department of Physics, University of Oslo, P.O. Box 1048 Blindern, NO-0316 Oslo, Norway
[5]Departments of Geosciences and Physics, The Njord Centre, University of Oslo, Oslo, Norway
[*]Corresponding author: Shahar Ben-Zeev, shahar.benzeev@mail.huji.ac.il

Supplementary Note 1. Terminology

Some commonly used terms in the soil liquefaction literature do not uniquely signify the physical processes that underlie observations. Here, we address two terminology issues that emerge from the current research.

Drained, partially drained and undrained

Experimental observations of liquefaction accompanied by a syn-shaking soil settlement [1,2], and experimental setups with boundary conditions that allow fluid flow [2–4] were denoted in previous studies as *partially drained*. In the current study, we refer to these settings as showing a *drained* response if $\mathrm{De} \ll 1$, as explained below.

The non-dimensional analysis, equation (2) in the main text and the non-dimensional Deborah number, De, equation (3) in the main text, define competing coupled grain-fluid effects in response to pore space changes as a function of the fluid ability to drain at the timescale of change. These competing effects lead to two end-member responses [5–7]. The first end-member occurs when $\mathrm{De} \gg 1$, fluid cannot flow out of or into a changing pore space during the change, and the pore pressure response is undrained. The second end-member occurs when $\mathrm{De} \ll 1$, as in our simulations and experiments. Here, fluid has sufficient time to flow in and out of the changing pore space, the fluid may be considered as incompressible, and the pore pressure response is drained. We propose that partially-drained (or partially-undrained) should be reserved for a case where $\mathrm{De} = \mathcal{O}(1)$. This terminology choice is consistent with several previous studies [5,6,8].

The choice of some studies to refer to $\mathrm{De} \ll 1$ as partially drained might reflect the dominance of the undrained paradigm for liquefaction triggering, where 'partially' is added to reconcile observations of fluid flow simultaneously with high pore fluid pressure and liquefaction. However, the coexistance of flow and pressurization is inherent to Darcy's law, which relates interstitial fluid flow (drainage) to fluid pressure gradients and thus to non-hydrostatic, elevated pore pressure.

Liquefaction and fluidization

The term *liquefaction* is used to describe a rheological change from a solid-like to a fluid-like soil behavior. The term *fluidization* is more commonly used when the rheological change is imposed by an externally-sourced fluid flux that enters through a (lower) boundary and via its upward-flow supports and lifts the grains [9,10].

We believe that the term *liquefaction* should be used to describe the dynamics emerging here in the drained simulations and experiments for several reasons. First, as demonstrated in figure (2) in the main text, the same field and experimental observations that are widely used to identify soil liquefaction, and which are commonly interpreted within the undrained liquefaction framework, are also observed here under drained conditions. Therefore, we suggest that the term *liquefaction* should be used to describe the observational phenomena, rather than a particular physical process. Second, the bottom boundaries of the simulations and experiments reported here are impermeable. No

external fluid flux crosses the boundary to induce the syn-seismic pressure gradient that support and liquefy grains above the compaction front. In drained shaking, the high pressure gradients that liquefy the soil emerge dynamically and in-situ due to the process of internal compaction, rendering the term *fluidization* inconsistent.

Supplementary Note 2. Liquefaction initiation timescale

There are two main candidate timescales for controlling t_i, the time to initiate drained liquefaction. The timescale of a single grain to fall down the fluid, and the time for an unloading front[11] to reach the liquefaction initiation depth.

We first explore the timescale associated with grain settlement and pressurization under drained conditions by considering the force balance over a single grain in the vertical direction:

$$\rho_s V \dot{u}_{sz} = \rho_s V g - \rho_f V g - \frac{V}{1-\phi}\frac{dP'}{dz}. \tag{S1}$$

According to equation (S1), the forces acting on such a grain are gravity, buoyancy and seepage by dynamic pore pressure gradient. To simplify the calculation, contact forces are not considered here. While contact forces are expected to be significant at the initial stages, when the pressure gradient rises, the grain becomes suspended and the magnitude of the contact forces likely drops.

Assigning the coupling between the pressure gradient and the grain's vertical velocity (Eq. 5 in the main text) in Eq. S1 and solving for the grain velocity with the initial condition $u_{sz}(t=0) = 0$:

$$u_{sz}(t) = \frac{\kappa}{\eta}(1-\phi)(\rho_s - \rho_f)g\ (1 - e^{-\frac{t}{\tau}}) \tag{S2}$$

where $\tau = \frac{\kappa\rho_s(1-\phi)}{\eta}$ is the exponential timescale for approaching terminal velocity. Assigning parameters for water and natural sand, $\eta = 10^{-3}$ Pa s, $\rho_s = 2600$ kg m^{-3}, $\phi = 0.5$ and $\kappa = 10^{-9} - 10^{-14}$ m^2, we find that $\tau \approx 10^{-3} - 10^{-8}$ s, is a fraction of a second. The pore pressure gradient follows a similar exponential evolution, with the same timescale, τ

$$\frac{dP'}{dz} = (1-\phi)(\rho_s - \rho_f)g\ (1 - e^{-\frac{t}{\tau}}). \tag{S3}$$

Our simulations and experiments show that both the grain settlement (downward) velocity and the pressurization approach their asymptotic values over a short timescale, yet much longer than τ. Thus the dynamics of a single grain falling in a fluid is probably not the rate-limiting process in initiating liquefaction. Our results indicate that t_i may be controlled by the descending unloading front that precedes the upward compaction front. Liquefaction initiation is only complete once this unloading front reaches the compaction front initiation depth, as seen in Fig. (6) in the main text and in supplementary note 6.

Supplementary Note 3. Compaction front initiation height

A careful analysis of the simulation results reveals that the height above the bottom boundary, from which the compaction front initiates, z_0, is not constant. z_0 is the thickness of the layer above the bottom wall that remains non-liquefied through shaking. Figure S1 shows that the initiation height of the compaction front correlates well with the imposed shaking frequency ($R^2 = 0.79$), where under higher input frequency, the compaction front starts closer to the bottom wall. We did not find another parameter that correlates as well with the initiation height ($PGV : R^2 = 0.06$; $PGA : R^2 = 0.33$, $e/T : R^2 = 0.03$).

Supplementary Note 4. Delayed fluid expulsion when the water level is below the surface

When the water level is located below the surface, different scenarios could emerge for the interactions between the compaction front, the water level and the surface. To define these interactions, we start by dividing the excited soil column to three regions: (1) below the compaction front, where the grain and fluid vertical velocities are approximately zero ($u_{sz}^{(1)} = u_{fz}^{(1)} \approx 0$), (2) above the compaction front and below the water level. Here, downward grain velocity is expressed by Eq. (6) in the main text as $u_{sz}^{(2)} = u_{sz_C} = (\kappa/\eta)(d\sigma_0/dz)$ $(d\sigma_0/dz < 0)$, and the upward fluid velocity is $u_{fz}^{(2)} = -((1-\phi_0)/\phi_0)\ u_{sz_C}$, and (3) above the water level where we assume that the downward grain velocity follows that of the second layer, $u_{sz}^{(3)} = u_{sz_C}$.

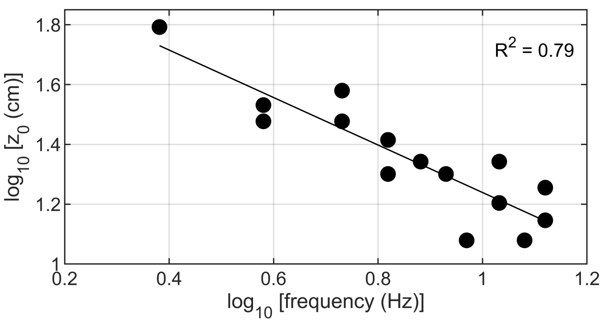

Figure S1: The height at which the compaction front initiates above the bottom boundary, z_0, as a function of imposed shaking frequency. The black line represents the best linear fit ($Y = -0.79\ X + 2$, $R^2 = 0.79$).

Next, we define the time dependent height of the top boundaries of the three regions with respect to the layer's base: (1) the height of the compaction front is expressed as $z_{\text{front}}(t) = z_0 + u_{\text{front}} \cdot t$, where u_{front} is defined in Eq. (4) in the main text and $z_0 = z_{\text{front}}(t = 0)$, (2) the height of the water table is $WL(t) = WL_0 + u^{(2)}_{fz} \cdot t$, where $WL_0 = WL(t = 0)$, and (3) the height of the soil layer is $h(t) = H + u^{(3)}_{sz} \cdot t$, where $H = h(t = 0)$, and $u^{(3)}_{sz}$ is negative.

A competition between the earthquake's duration, t_{EQ}, the time for the front to arrive to the water level, t^{WL}_{front}, and the time for the water level to arrive to the surface, $t^{surface}_{WL}$, controls the potential for different observations, where:

$$t^{WL}_{front} = \frac{WL_0 - z_0}{u_{\text{front}} - u^{(2)}_{fz}} = \frac{WL_0 - z_0}{\frac{\kappa}{\eta}\frac{d\sigma_0}{dz}\left(\frac{\phi_0 - 1}{\phi_0 - \phi_c} + \frac{1-\phi_0}{\phi_0}\right)}, \tag{S4}$$

and

$$t^{surface}_{WL} = \frac{H - WL_0}{u^{(2)}_{fz} - u^{(3)}_{sz}} = \frac{H - WL_0}{-\frac{\kappa}{\eta}\frac{d\sigma_0}{dz}\left(\frac{1-\phi_0}{\phi_0} + 1\right)}. \tag{S5}$$

When the water level is initially close to surface, as is the case in coastal and riverbank sediments, then for a wide range of parameter sets, $t^{surface}_{WL} < \min(t_{EQ}, t^{WL}_{front})$, and fluid will seep co-seismically at the surface, making liquefaction easily detectable. For example, consider a 10 meter deep soil column ($H = 10$ m) with $\kappa = 2\times10^{-9}$ m^2, an initial water level that is located 30 cm below the surface ($WL_0 = 9.7$ m), and excitation that induces $\Delta\phi = 9 \times 10^{-3}$, the water will seep at the surface after $t^{surface}_{WL} \simeq 7$ seconds, while the whole liquefaction event will last for $t^{WL}_{front} \simeq 8.5$.

However, if the permeability is lower, the excitation is weaker (leading to a smaller $\Delta\phi$), or the initial water level is deeper, then $t^{WL}_{front} < t^{surface}_{WL}$, and the liquefaction event may terminate before water reaches and seeps out of the surface. For example, consider the parameters used above, but when the initial water level is 2 meters below the surface ($WL_0 = 8$ m), than the event will terminate after $t^{WL}_{front} \simeq 14$ seconds without a co-seismic seepage, since $t^{surface}_{WL} \simeq 90$ seconds.

Supplementary Note 5. High gravity experiments and simulations support depth-independent triggering of drained liquefaction

The theory and inferred dynamics of drained liquefaction described in this study are scale-less. To further demonstrate that, we reanalyze centrifuge experiments and high gravity discrete element method simulation, representing greater layers' depth and higher stresses with respect to the simulations and experiments described in the main text. Notably, reanalysis of previously published high gravity experiments and simulations is not trivial since the duration of the imposed shaking is commonly short, limiting observations of the co-seismic dynamics. Furthermore, the elaborated recent LEAP project[12] was disqualified from the current reanalysis because of the imposed initial soil layer surface topography, designed to study lateral spreading, which hinders the identification of compaction front dynamics.

Ref.[2] conducted centrifuge experiments of shaken saturated soil under 50 g, representing a 12.25 meters thick soil in prototype scale. The authors calculated the drainage conditions using a De number-like framework and concluded that the soil layer was *"partially drained"* during liquefaction (see Supplementary Note 1) and that *"fluid flow will very quickly spread localized pressure differences, making an undrained approximation inappropriate for the interpretation of the experimental results"*[2].

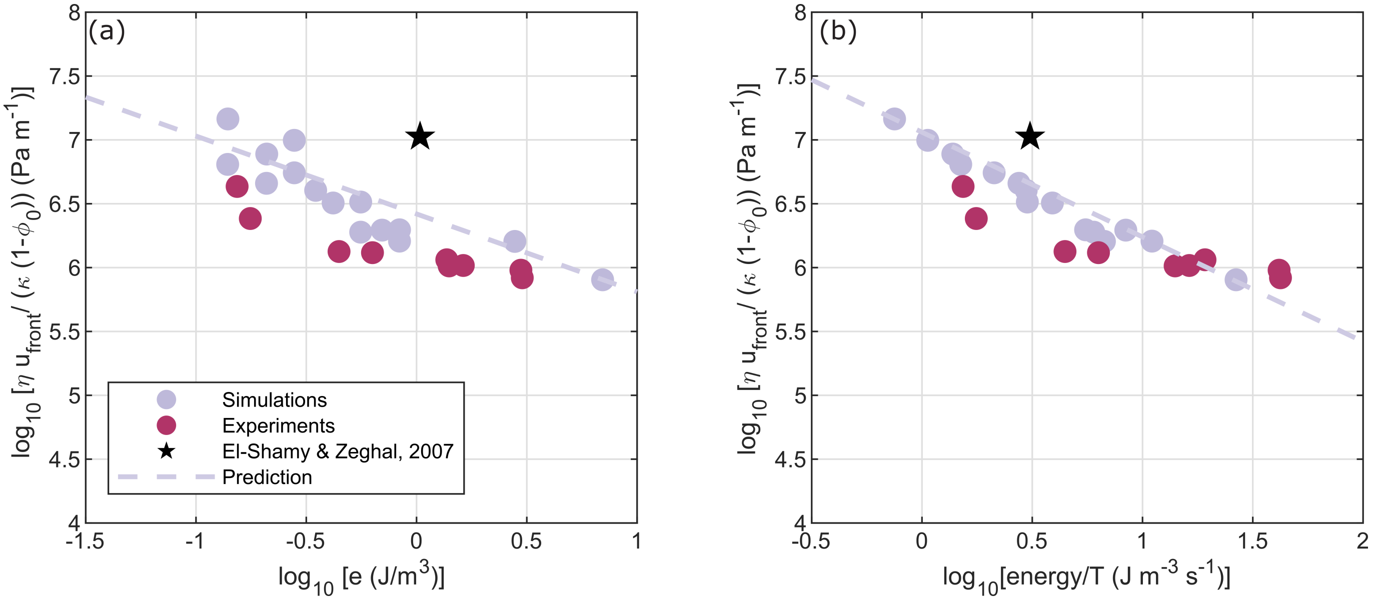

Figure S2: Reproduction of Fig.(3)d and inset (of the main text) with the data of ref.[13] plotted as black stars. (a) Scaled compaction front velocity vs. seismic energy density. (b) Scaled compaction front velocity vs. the rate of seismic energy density. The stars represent data extracted from a 30 g simulation (corresponding to a 5.2 meters deep layer in prototype scale)[13].

Two of the observations described in ref.[2] are in line with the model proposed in the current study: (i) A very short timescale for complete pressurization since the onset of shaking ($2-4$ shear cycles) and (ii) An observation of co-seismic soil settlement at a rate that roughly corresponds to the theoretical prediction of equation (6) in the main text. Accounting for the authors' evaluation of their layer permeability, viscosity, and initial porosity, we estimate the co-seismic settlement rate, based on equation (6) in the main text as $u_{sz_C} \approx 0.9$ mm s^{-1}. Digitization of Figure 6 in ref.[2] suggests a similar order of magnitude for their co-seismic settlement of $u_{sz_C} \approx 0.38 - 0.44$ mm s^{-1}. Considering the uncertainty in the evaluation of the permeability and initial porosity, the factor of $\sim$2 difference between the prediction and measurement is small.

A second study to which we compare our theory is Ref.[13], which conducted 3D numerical simulations of a coupled grain and fluid system. Their simulation, under a high gravity of 30 g, represents an unconfined 5.2 meters thick soil layer in prototype scale. We evaluate the De number (Eq. (3) in the main text) for this simulation (with model scale parameters) to be De $= 0.004 \ll 1$, indicating drained conditions. The evaluation of the De number was achieved by using a permeability of $\kappa_0 = 1.2 \times 10^{-7}$m^2 (based on the first term in their equation (3)), a reported porosity of $\phi_0 = 0.41$, and an estimated front thickness of 20 grain diameter.

We further evaluate the co-seismic compaction front velocity in the simulation of ref.[13] by using the pore pressure ratio at the deepest point of measurement (figure 20 in ref.[13]). The rate of co-seismic pore pressure ratio decline at that depth is expected to be equal to the compaction front velocity times the depth (see equations 26-27 in ref.[7]), and is evaluated to be $u_{\text{front}} \approx 0.028$ m s^{-1}. The maximal seismic energy density applied in the simulation was $e = 1.03$ J m^{-3} and the maximal rate of seismic energy density was $e/T = 3.11$ J m^{-3} s^{-1}. Fig. S2 shows the inferred scaled compaction front velocity as a function of the maximal seismic energy density (panel a) and rate of seismic energy density (panel b) as black stars, showing good agreement with the general trend and prediction developed in the current study.

While the agreement between the results of refs.[2,13] and the theory developed in the current study demonstrate that drained liquefaction triggering under low seismic energy density is feasible under several meters depth and high stresses, we note that a more rigorous comparison is needed.

Supplementary Note 6. Grain velocity maps

Here, we show grain velocity maps (of simulations, see Table 2 in the main text), normalized by the theoretical terminal velocity, following Eq. (6) in the main text. The black lines depict the compaction front location over time ($z_{\text{front}}(t)$) and the red markers show the initial and final locations used for the calculation of the front velocity (see Methods in the main text). The velocity signal is smoothed following the procedure described in the Methods section in the

main text. For each simulation, the raw normalized velocity values are presented on the left panel, and the values subjected to a $0-1$ threshold are shown on the right panel. Grains settlement at an approximately constant terminal velocity in shown in yellow, and grains that are approximately vertically stagnant are in blue. The vertical velocity of grains is proportional to the pore pressure gradients (Eq. (5) in the main text), hence yellow areas in the maps means complete liquefaction. In some of the simulations, the fast downward propagating "unloading front" which precedes the "compaction front", can be observed.

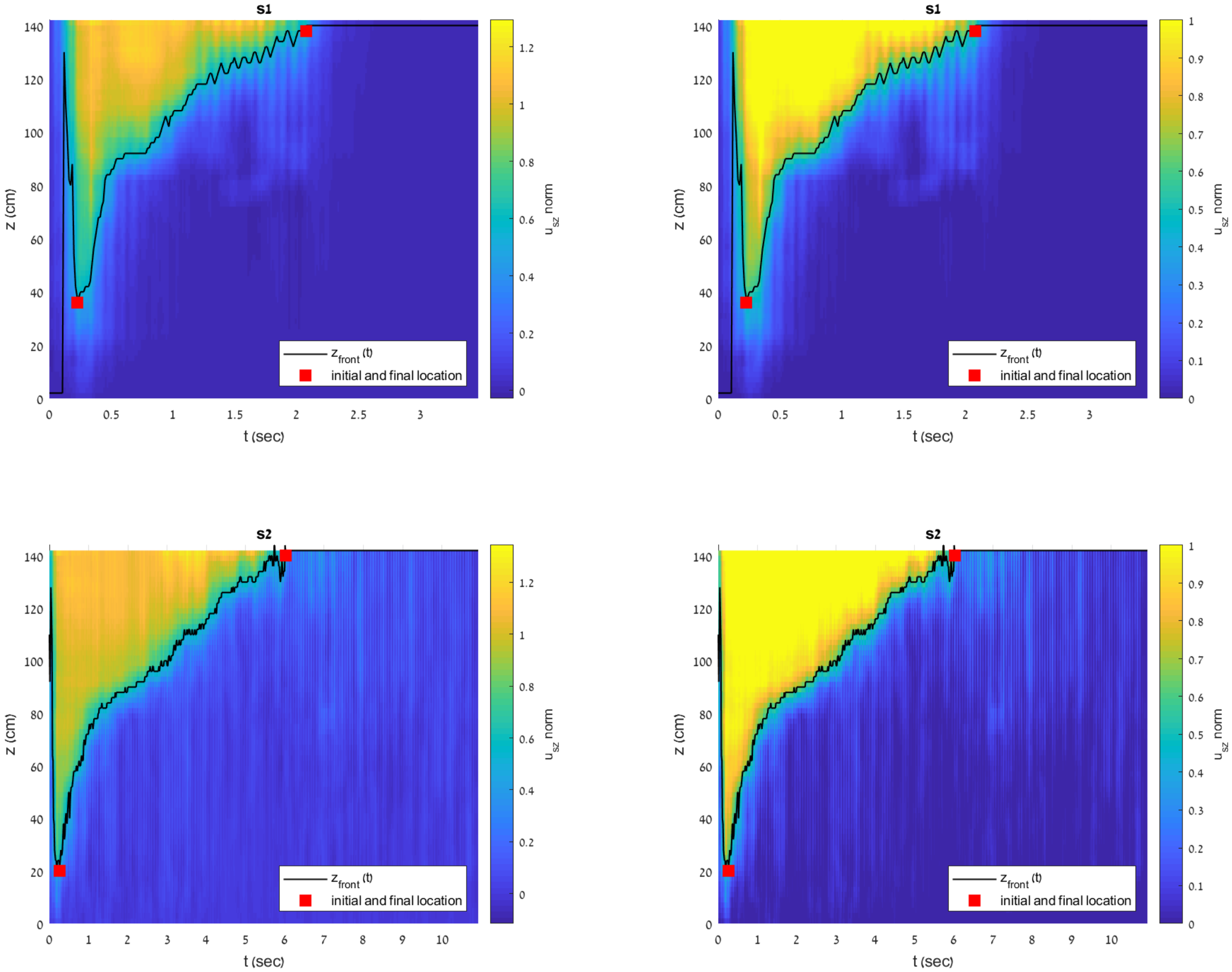

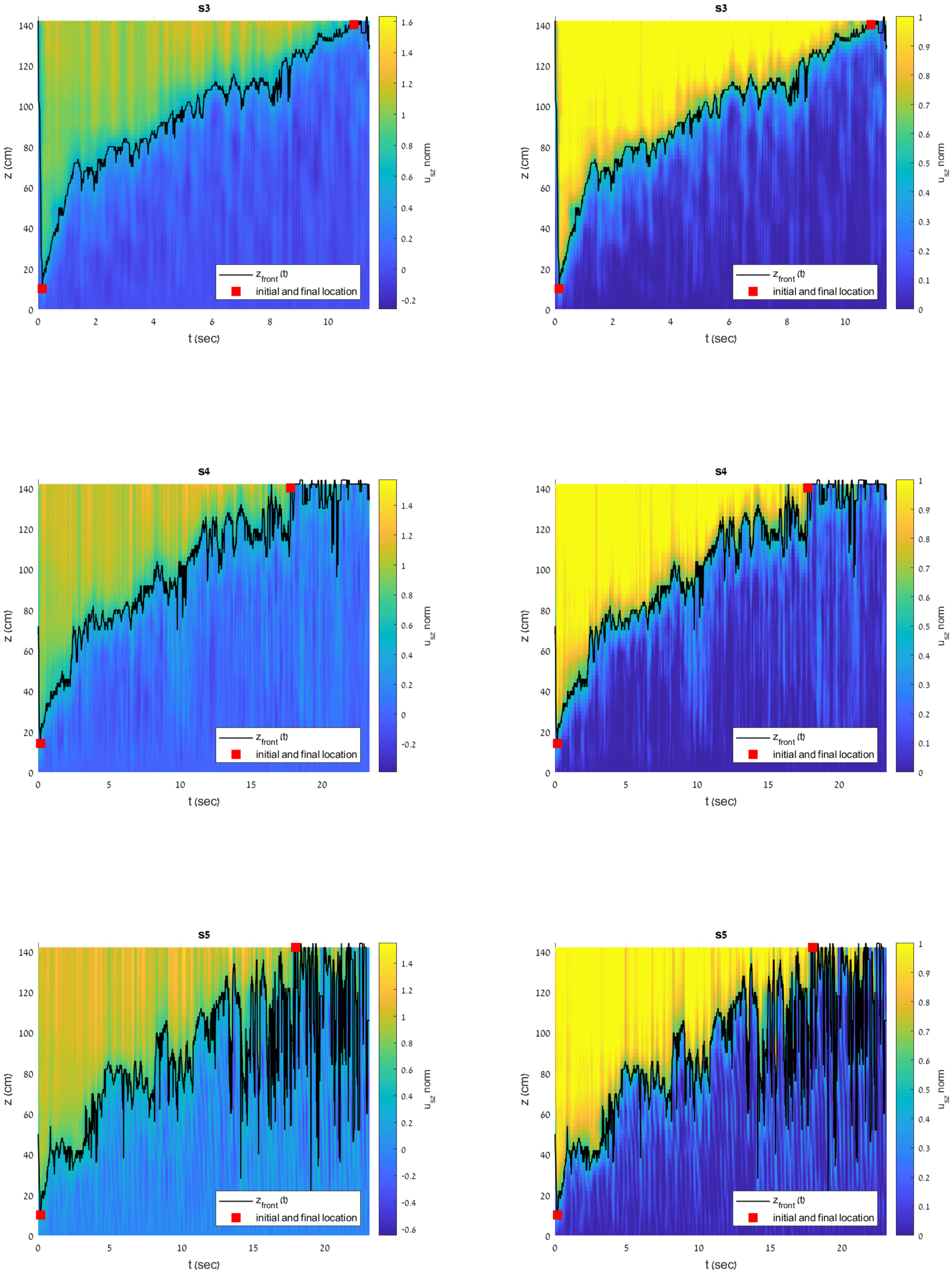
S3
S3
S4
S4
S5
S5
z (cm)
t (sec)
u_sz norm
z_front (t)
initial and final location

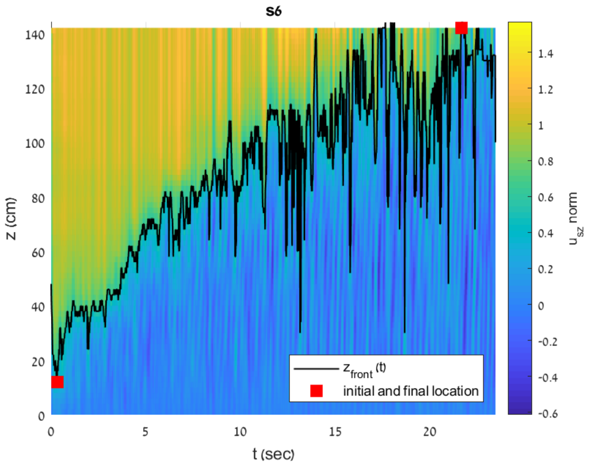
S6
z (cm)
t (sec)
u_{sz} norm
z_{front} (t)
initial and final location

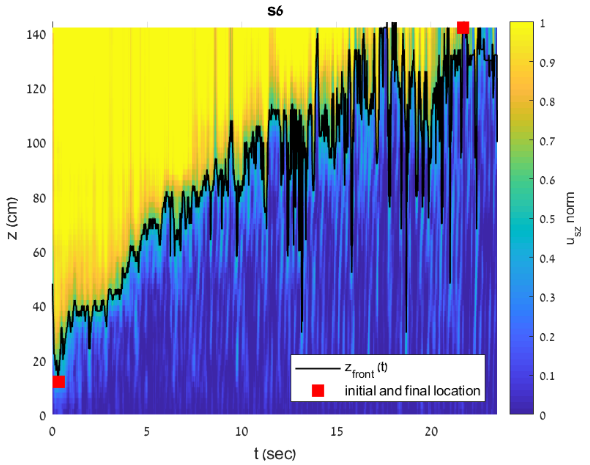
S6
z (cm)
t (sec)
u_{sz} norm
z_{front} (t)
initial and final location

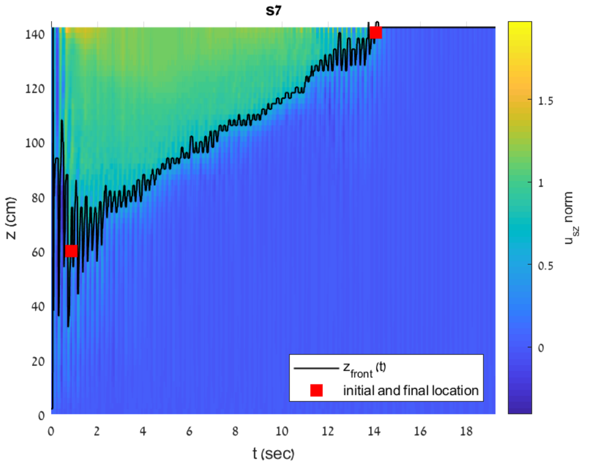
S7
z (cm)
t (sec)
u_{sz} norm
z_{front} (t)
initial and final location

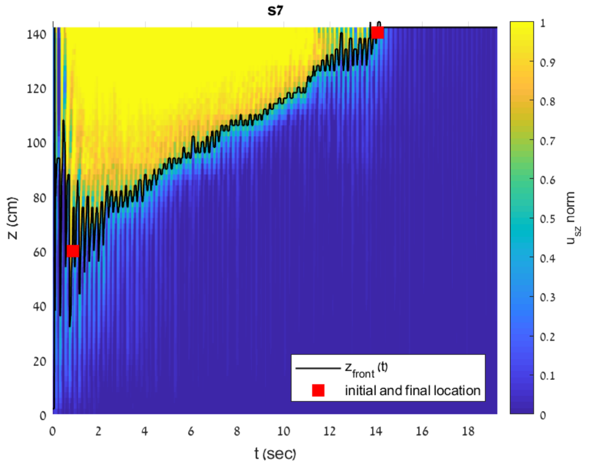
S7
z (cm)
t (sec)
u_{sz} norm
z_{front} (t)
initial and final location

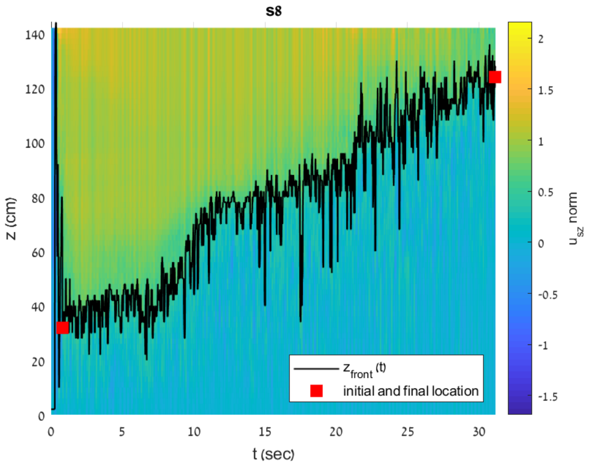
S8
z (cm)
t (sec)
u_{sz} norm
z_{front} (t)
initial and final location

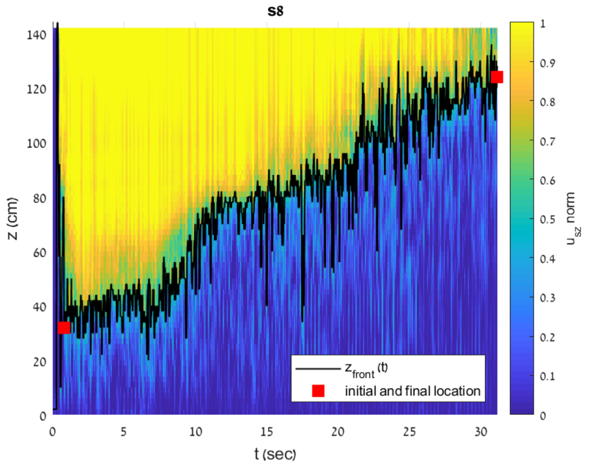
S8
z (cm)
t (sec)
u_{sz} norm
z_{front} (t)
initial and final location

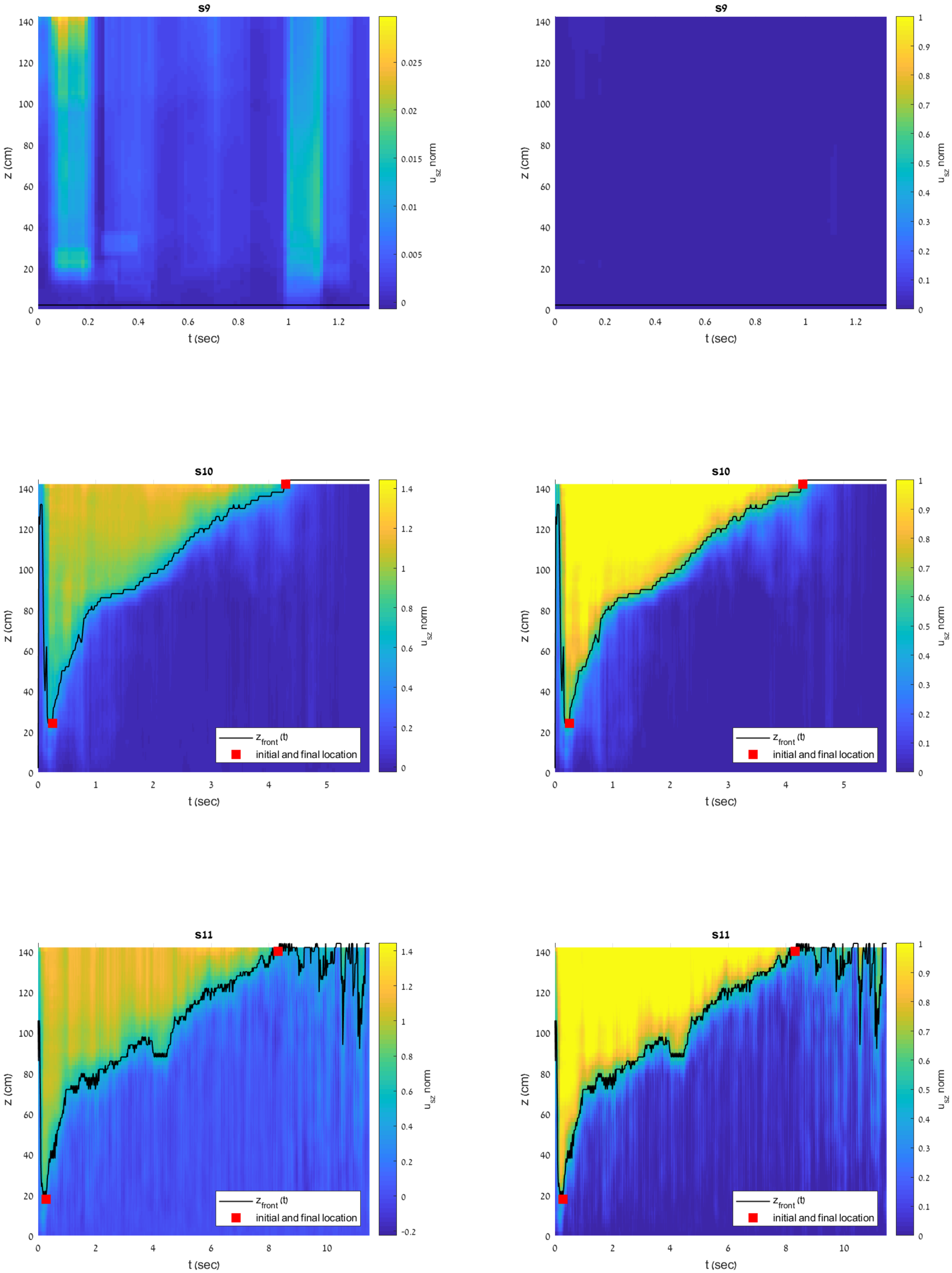

S9
S9
S10
S10
S11
S11
z (cm)
t (sec)
u_sz norm
z_front (t)
initial and final location

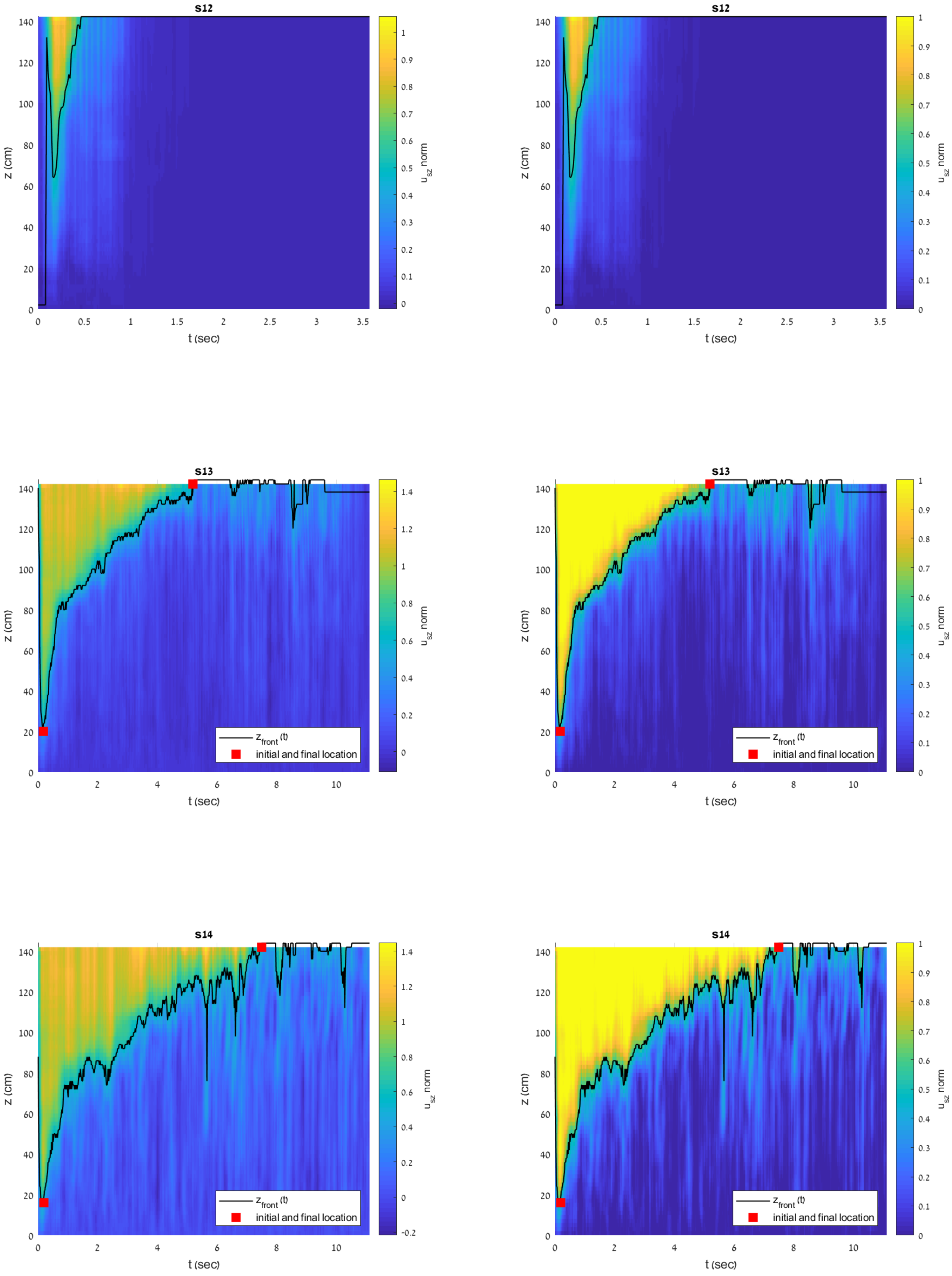

S12
z (cm)
t (sec)
u_sz norm
S12
z (cm)
t (sec)
u_sz norm
S13
z (cm)
t (sec)
u_sz norm
z_front (t)
initial and final location
S13
z (cm)
t (sec)
u_sz norm
z_front (t)
initial and final location
S14
z (cm)
t (sec)
u_sz norm
z_front (t)
initial and final location
S14
z (cm)
t (sec)
u_sz norm
z_front (t)
initial and final location

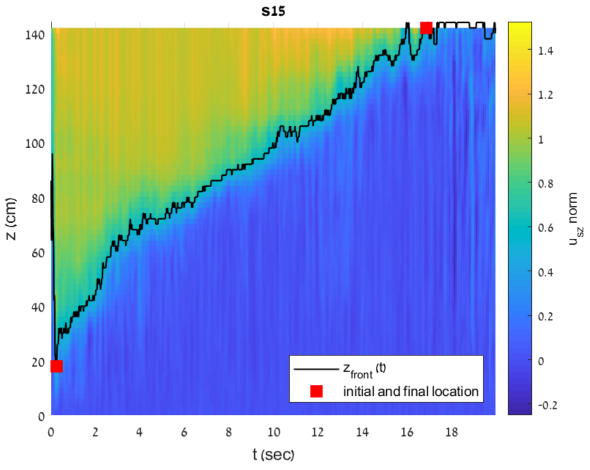
S15
z (cm)
t (sec)
u_sz norm
z_front (t)
initial and final location
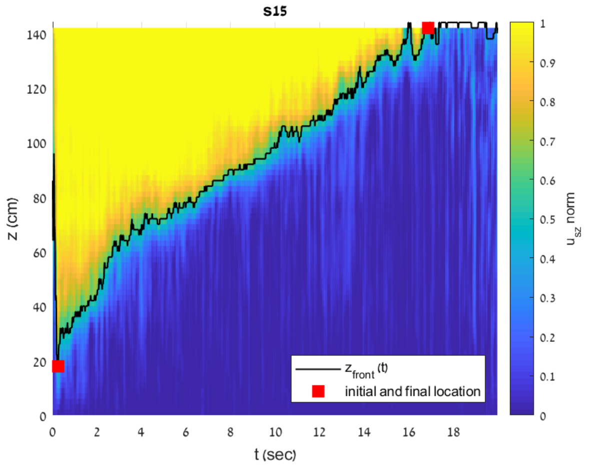
S15
z (cm)
t (sec)
u_sz norm
z_front (t)
initial and final location
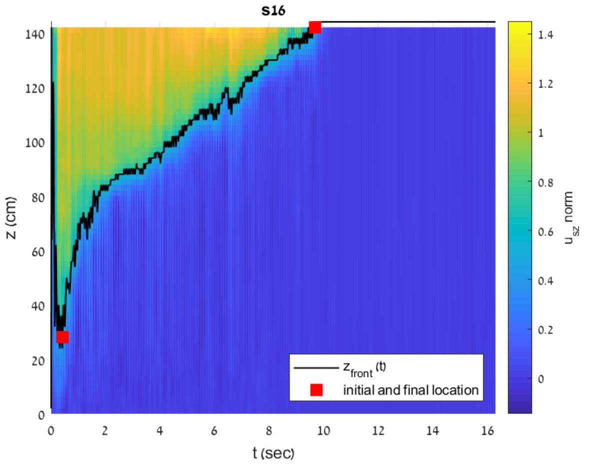
S16
z (cm)
t (sec)
u_sz norm
z_front (t)
initial and final location
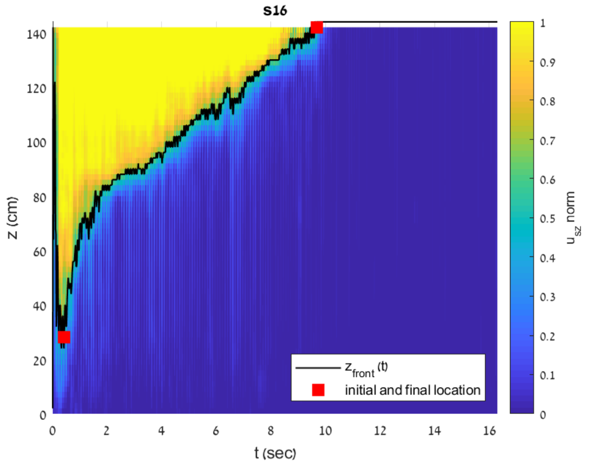
S16
z (cm)
t (sec)
u_sz norm
z_front (t)
initial and final location
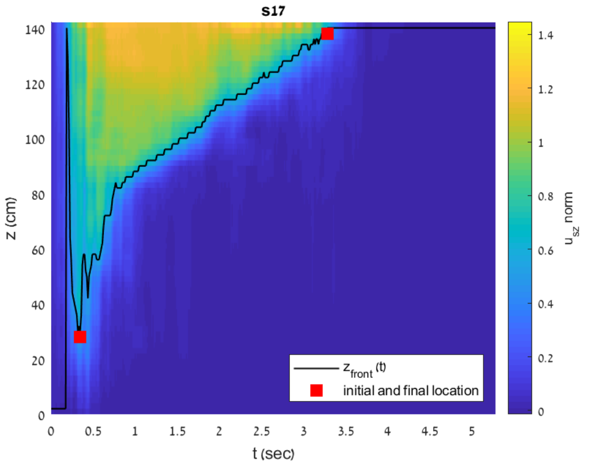
S17
z (cm)
t (sec)
u_sz norm
z_front (t)
initial and final location
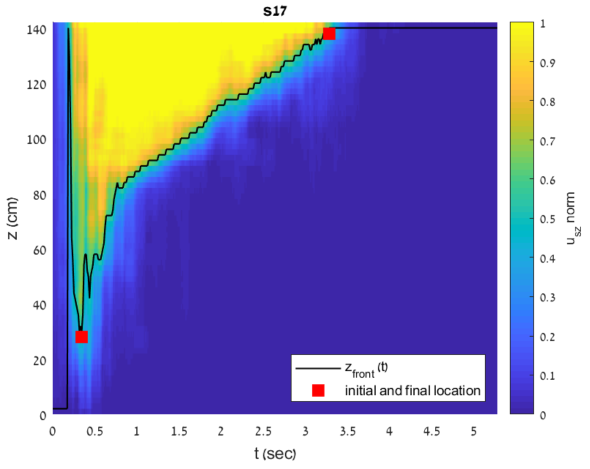
S17
z (cm)
t (sec)
u_sz norm
z_front (t)
initial and final location

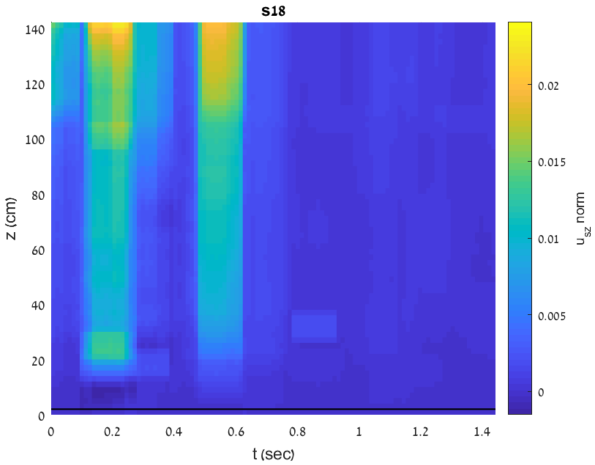
S18
z (cm)
t (sec)
u_sz norm

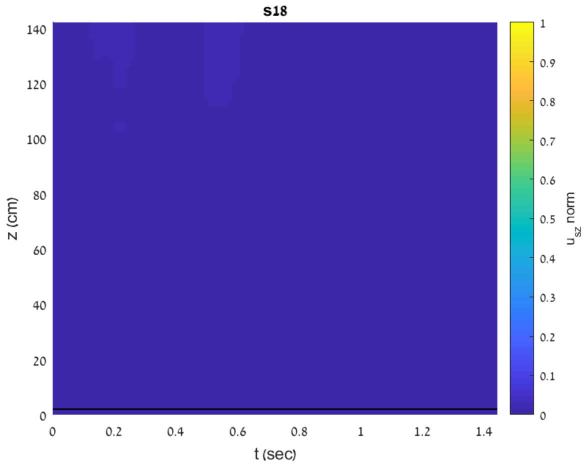
S18
z (cm)
t (sec)
u_sz norm

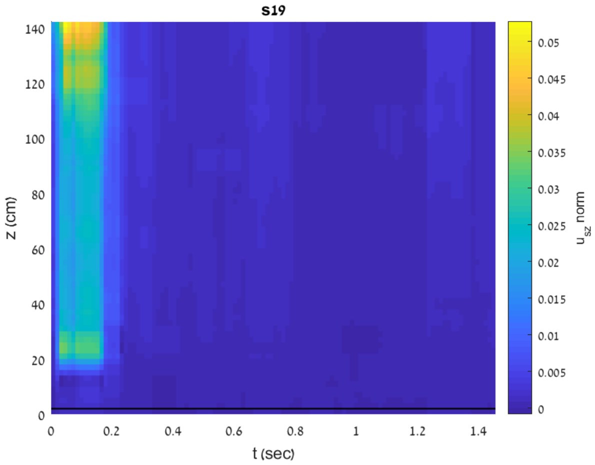
S19
z (cm)
t (sec)
u_sz norm

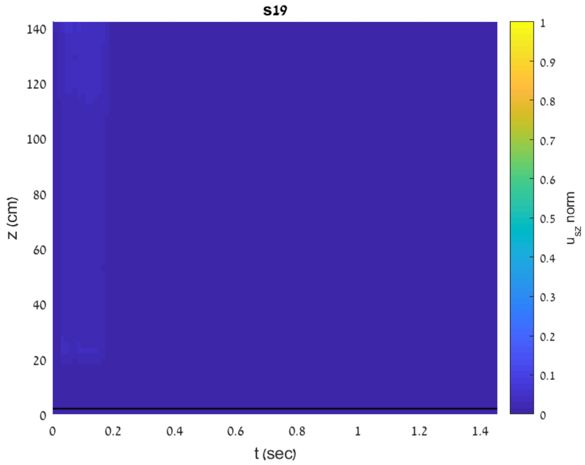
S19
z (cm)
t (sec)
u_sz norm

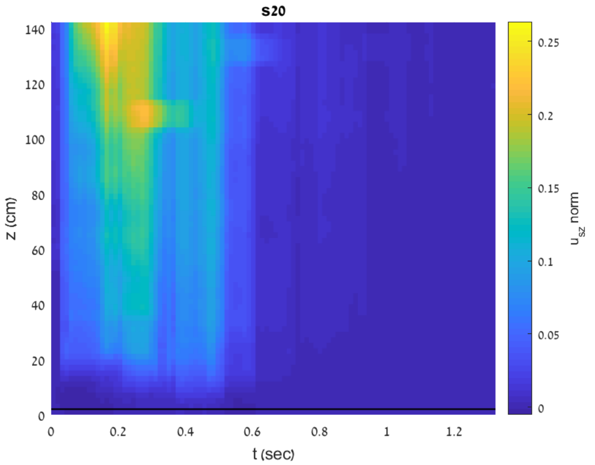
S20
z (cm)
t (sec)
u_sz norm

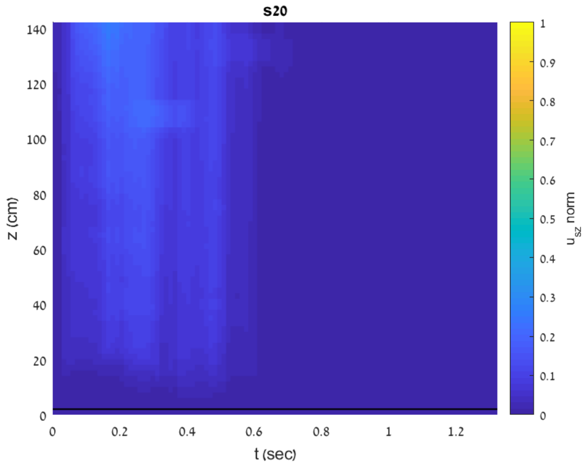
S20
z (cm)
t (sec)
u_sz norm

References

[1] Madabhushi, G. S. P. & Haigh, S. K. How well do we understand earthquake induced liquefaction? *Indian Geotechnical Journal* **42**, 150–160 (2012).

[2] Adamidis, O. & Madabhushi, G. Experimental investigation of drainage during earthquake-induced Experimental investigation of drainage during earthquake-induced liquefaction. *Geotechnique* **68**, 655–665 (2018).

[3] Kamai, R. & Boulanger, R. W. Single-element simulations of partial-drainage effects under monotonic and cyclic loading. *Soil Dynamics and Earthquake Engineering* **35**, 29–40 (2012). URL `http://dx.doi.org/10.1016/j.soildyn.2011.10.002`.

[4] Adamidis, O. & Anastasopulis, I. Cyclic liquefaction resistance of sand under a constant inflow rate. *Geotechnique* (2022).

[5] Goren, L., Aharonov, E., Sparks, D. W. & Toussaint, R. Pore pressure evolution in deforming granular material: A general formulation and the infinitely stiff approximation. *Journal of Geophysical Research: Solid Earth* **115**, 1–19 (2010).

[6] Goren, L., Aharonov, E., Sparks, D. W. & Toussaint, R. The mechanical coupling of fluid-filled granular material under shear. *Pure and Applied Geophysics* **168**, 2289–2323 (2011).

[7] Ben-Zeev, S., Aharonov, E., Toussaint, R., Parez, S. & Goren, L. Compaction front and pore fluid pressurization in horizontally shaken drained granular layers. *Physical Review Fluids* **054301**, 1–25 (2020).

[8] Lakeland, D. L., Rechenmacher, A. & Ghanem, R. Towards a complete model of soil liquefaction : the importance of fluid flow and grain motion. *Proceedings of the royal society* **470** (2014).

[9] McNamara, S., Flekkøy, E. G. & Måløy, K. J. Grains and gas flow: molecular dynamics with hydrodynamic interactions. *Physical review E* **61**, 4054—-4059 (2000).

[10] Haigh, S., Eadington, J. & Madabhushi, S. P. G. Permeability and stiffness of sands at very low effective stresses. *Geotechnique* **62**, 69–75 (2012).

[11] Florin, V. & Ivanov, P. Liquefaction of Saturated Sandy Soils. In *Proceedings of the 5th international conference on soil mechanics and foundation engineering*, 107–11 (1961).

[12] Kutter, B. L., Manzari, M. T. & Zeghal, M. *Model Tests and Numerical Simulations of Liquefaction and Lateral Spreading - LEAP-UCD-2017* (Springer Open, 2020).

[13] El Shamy, U. & Zeghal, M. A micro-mechanical investigation of the dynamic response and liquefaction of saturated granular soils. *Soil Dynamics and Earthquake Engineering* **27**, 712–729 (2007).
